# Dissecting causal associations of type 2 diabetes with 111 types of ocular conditions: a Mendelian randomization study

**DOI:** 10.3389/fendo.2023.1307468

**Published:** 2023-11-22

**Authors:** Rumeng Chen, Shuling Xu, Yining Ding, Leyang Li, Chunxia Huang, Meihua Bao, Sen Li, Qiuhong Wang

**Affiliations:** ^1^ School of Life Sciences, Beijing University of Chinese Medicine, Beijing, China; ^2^ Department of Endocrinology, Guang’anmen Hospital, China Academy of Chinese Medical Sciences, Beijing, China; ^3^ School of Stomatology, Changsha Medical University, Changsha, China; ^4^ The Hunan Provincial Key Laboratory of the TCM Agricultural Biogenomics, Changsha Medical University, Changsha, China; ^5^ Hunan Key Laboratory of the Research and Development of Novel Pharmaceutical Preparations, School of Pharmaceutical Science, Changsha Medical University, Changsha, China

**Keywords:** type 2 diabetes, ocular diseases, diabetes complications, Mendelian randomization, causal association

## Abstract

**Background:**

Despite the well-established findings of a higher incidence of retina-related eye diseases in patients with diabetes, there is less investigation into the causal relationship between diabetes and non-retinal eye conditions, such as age-related cataracts and glaucoma.

**Methods:**

We performed Mendelian randomization (MR) analysis to examine the causal relationship between type 2 diabetes mellitus (T2DM) and 111 ocular diseases. We employed a set of 184 single nucleotide polymorphisms (SNPs) that reached genome-wide significance as instrumental variables (IVs). The primary analysis utilized the inverse variance-weighted (IVW) method, with MR-Egger and weighted median (WM) methods serving as supplementary analyses.

**Results:**

The results revealed suggestive positive causal relationships between T2DM and various ocular conditions, including “Senile cataract” (OR= 1.07; 95% CI: 1.03, 1.11; *P*=7.77×10^-4^), “Glaucoma” (OR= 1.08; 95% CI: 1.02, 1.13; *P*=4.81×10^-3^), and “Disorders of optic nerve and visual pathways” (OR= 1.10; 95% CI: 0.99, 1.23; *P*=7.01×10^-2^).

**Conclusion:**

Our evidence supports a causal relationship between T2DM and specific ocular disorders. This provides a basis for further research on the importance of T2DM management and prevention strategies in maintaining ocular health.

## Introduction

Type 2 diabetes mellitus (T2DM) accounts for approximately 90% of the global population of 537 million individuals with diabetes mellitus (DM), predominantly affecting individuals over the age of 55 (1). The prevalence of T2DM is rising due to accelerated global aging, and it is projected that the worldwide diabetic population will reach 783 million by 2045 (2). DM can give rise to a range of complications, characterized by elevated disability and mortality rates (3–8). Visual impairment stands out as a particularly severe complication (9).

A systematic review and meta-analysis of global population-based eye disease surveys conducted from 1990 to 2020 established that cataract, glaucoma, age-related macular degeneration (AMD), and diabetic retinopathy (DR) are the primary causes of blindness. Importantly, early detection and timely intervention can prevent these conditions (10). It is widely recognized that there is a strong association between DM and the development of DR, with approximately one-third of diabetic patients affected by this condition (11). Furthermore, DM has been linked to several other significant visual impairments worldwide, including cataract, AMD, and glaucoma (12). For instance, a meta-analysis indicated that DM was associated with an increased risk of AMD (13), while two other studies reported a 50% reduction in AMD prevalence among patients with DM (14, 15). Moreover, there are conflicting reports regarding the association between diabetes and glaucoma. While a meta-analysis suggested that diabetes increased the prevalence of glaucoma (16), another study revealed that DM conferred a protective effect against the development of glaucomatous optic nerve damage in patients with primary open-angle glaucoma (POAG) (17). Hence, the existing research on DM and ocular-related diseases remains inconclusive. The primary reason for these conflicting findings is that most studies examining the relationship between diabetes and ocular-related diseases are observational, making it difficult to exclude the effects of confounding variables and reverse causality.

Mendelian Randomization (MR) analysis is an epidemiological approach that utilizes genetic variants associated with a specific exposure as instrumental variables (IVs). Its primary objective is to evaluate potential causal relationships between the exposure and outcome measures (18). The methodologies employed in MR analysis are grounded in Mendel’s second law, which states that alleles are randomly allocated. This characteristic, irrespective of the individual’s illness status, helps mitigate biases induced by confounding factors and reverse causality. The objective of this study is to employ the MR method to examine the causal relationship between T2DM and 111 types of ocular conditions. Furthermore, it seeks to establish a theoretical basis for the prevention and treatment of eye diseases in individuals with diabetes.

## Methods

### Study design

Based on the dataset acquired from the genome-wide association study (GWAS), we identified specific single nucleotide polymorphisms (SNPs) that exhibited significant associations with T2DM as the exposure variable. We utilized these SNPs as IVs and employed a MR analysis to evaluate the causal association between T2DM and the aforementioned ocular diseases.

### Data sources

The data for the exposure variable (T2DM) (including 74,124 T2DM cases and 824,006 controls of European ancestry) was obtained from Anubha Mahajan et al.’s study (19), while data for the outcome variables were obtained from all 111 eye-related diseases (detailed information can be found in **Supplementary Table 1**) available in the FinnGen database, which is a comprehensive biomedical research project conducted in Finland (20).

### Selection of IVs

We implemented specific criteria for the selection of IVs in our MR analysis. These criteria included: (1) establishing a significant genomic-level association between the IVs and the exposure (*P* < 5.00×10^-8^), (2) ensuring the independent selection of IVs by clumping within a 10 Mb window and minimizing linkage disequilibrium (R^2^ < 0.001), and (3) setting a minimum minor allele frequency (MAF) threshold of 0.01. We employed F-statistics to evaluate the strength of the IVs, considering values greater than 10 as indicative of a lower probability of weak instrument bias (21).

### MR analysis

Among the three MR methods employed in this study, the inverse variance-weighted (IVW) method was the primary approach. IVW primarily evaluates the results by aggregating the MR effect estimates for each individual SNP. For the weighted median (WM) method to be applicable, it is necessary for the valid variable to constitute a minimum of 50%. Furthermore, an intercept term is employed by MR-Egger to assess potential pleiotropy.

### Sensitivity analysis

To detect and eliminate potential outliers, we employed pleiotropy-corrected data from MR-PRESSO. We assessed heterogeneity using Cochrane Q-values. Furthermore, we employed the Leave-one-out method by removing one SNP at a time and reanalyzing whether the remaining SNPs significantly impacted the results. Causal estimates were obtained by calculating odds ratios (ORs) and their corresponding 95% confidence intervals (CIs). To handle multiple comparisons, we applied a false discovery rate (FDR) of 5%. Causal associations were considered significant if they survived FDR correction, but suggestive associations were also discussed in our study. The two-sample MR software package in R was utilized for conducting all MR analyses.

## Results

### Assessment of the IVs

We identified 184 SNPs from T2DM as IVs, with F statistic values ranging from 29.47 to 1393.78 (**Supplementary Table 2**).

### Results of the MR analysis

**Figure 1:**
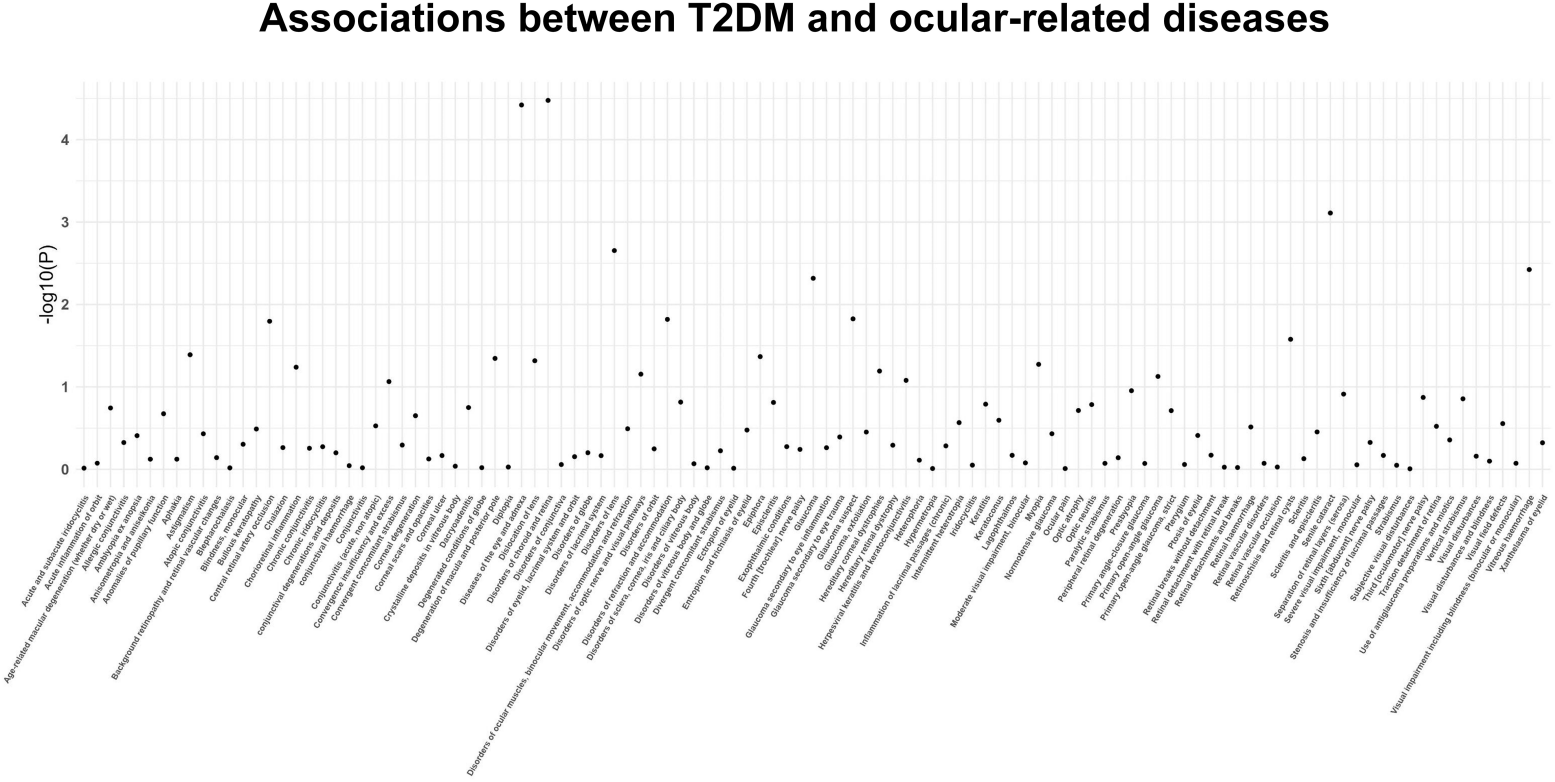
The *P*-value distribution of associations between type 2 diabetes mellitus and 111 ocular-related diseases in the Mendelian randomization analysis.

**Figure 2:**
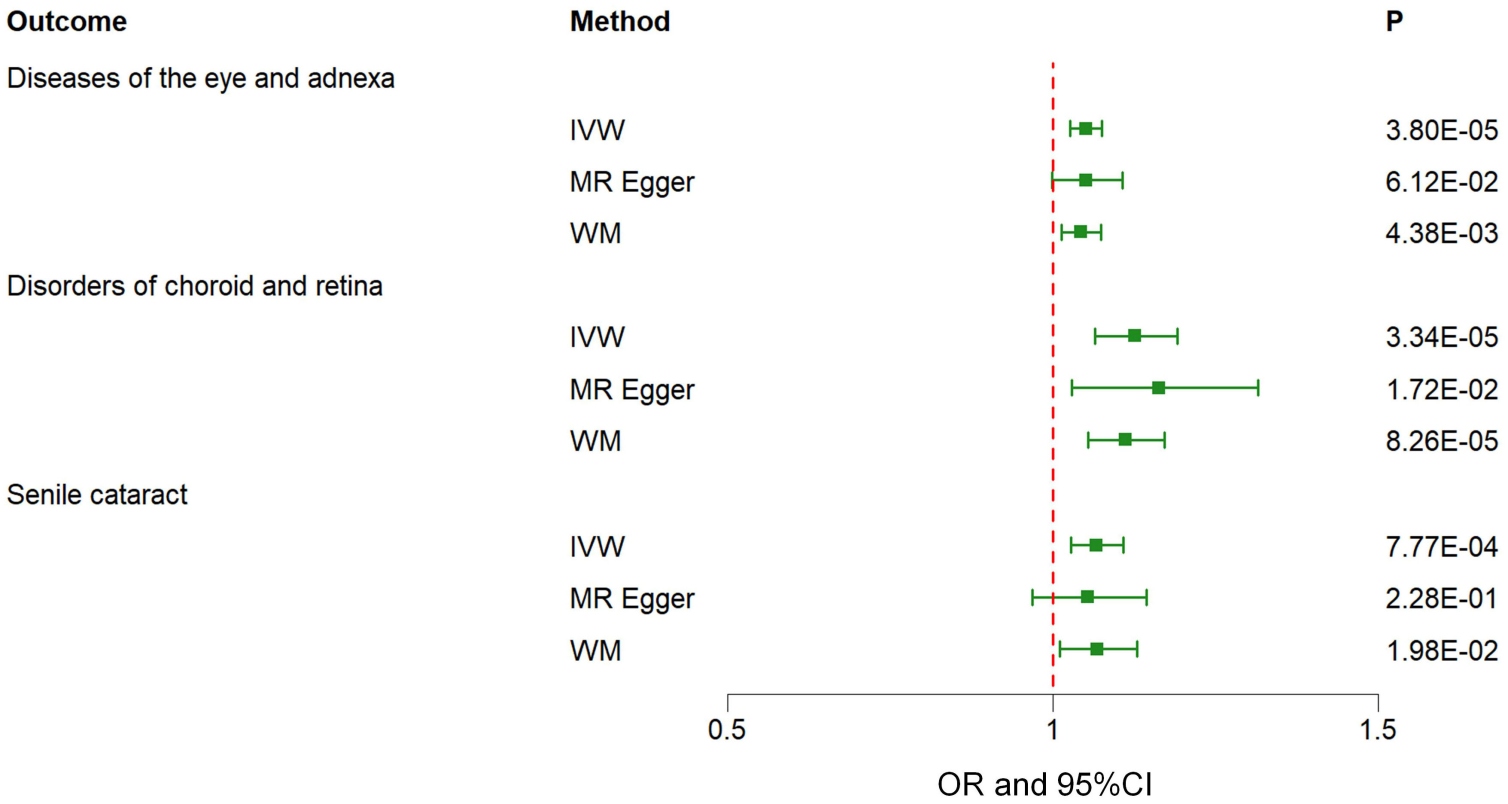
Associations between genetically predicted type 2 diabetes mellitus and various ocular-related diseases examined by three MR methods. MR, Mendelian randomization; IVW, inverse-variance weighted; WM, weighted median; CI, confidence interval.

**Figure 3:**
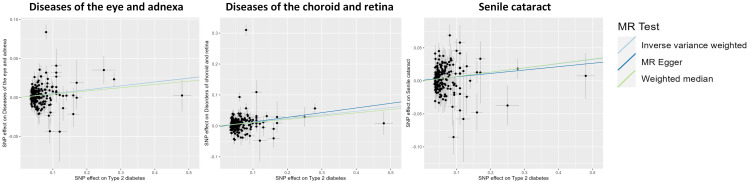
Scatter plot showing the causal effects of type 2 diabetes mellitus on various ocular-related diseases. SNP, single nucleotide polymorphism.

The findings of the IVW method indicated a potential causal association between T2DM and various ocular-related diseases, including “Vitreous haemorrhage” (OR= 1.21; 95% CI: 1.06, 1.38; *P*=3.77×10^-3^), “Senile cataract” (OR= 1.07; 95% CI: 1.03, 1.11; *P*=7.77×10^-4^), “Glaucoma” (OR= 1.08; 95% CI: 1.02, 1.13; *P*=4.81×10^-3^), and “Disorders of optic nerve and visual pathways” (OR= 1.10; 95% CI: 0.99, 1.23; *P*=7.01×10^-2^) (**Figures 1**, **2**; **Supplementary Table 3**). Furthermore, significant associations persisted between T2DM and diseases of the eye and adnexa, disorders of choroid and retina, and senile cataract even after adjusting for multiple comparisons. Using the MR-Egger and WM approaches, the relationships between T2DM and these ocular-related diseases had the same direction (**Figure 2**; **Supplementary Table 3**). **Figure 3** displays the scatter plot illustrating the causal relationships between T2DM and these ocular-related diseases.

### Results of the sensitivity analysis

**Figure 4:**
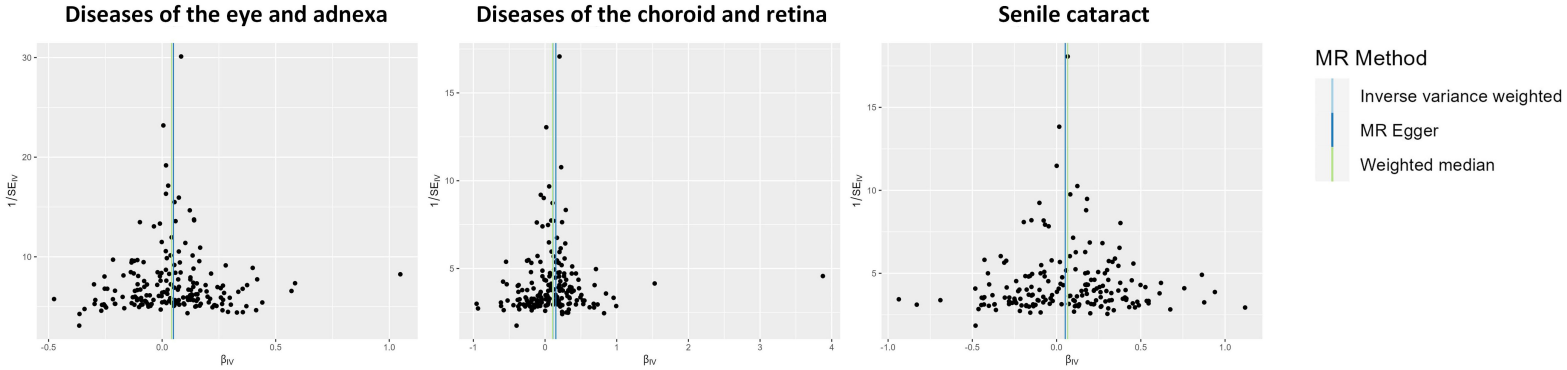
Funnel plot indicating the causal associations of type 2 diabetes mellitus on various ocular-related diseases. SNP, single nucleotide polymorphism; IV, instrumental variable; SE, standard error.

The potential heterogeneity was evaluated (**Figure 4**; **Supplementary Table 4**). The findings presented in **Supplementary Figure 1** indicate that most individual SNPs had minimal impact on the results during the leave-one-out analysis. The MR-Egger method did not detect the presence of horizontal pleiotropy (**Supplementary Table 5**). Despite the MR-PRESSO analysis identifying several outliers in the results, it did not significantly alter the outcomes after correction (**Supplementary Table 6**).

## Discussion

In our MR analysis involving 111 ocular diseases, we identified potential causal associations between T2DM and various ocular-related diseases, such as diseases of the eye and adnexa, disorders of choroid and retina, vitreous haemorrhage, senile cataract, glaucoma, and disorders of optic nerve and visual pathways. After applying multiple corrections, the causal relationship between T2DM and diseases of the eye and adnexa, senile cataract, as well as disorders of choroid and retina, remained statistically significant. According to the definition provided by FinnGen, diseases of the eye and adnexa encompass disorders of choroid and retina (including any disease or disorder of the retina), glaucoma (characterized by increased ocular pressure due to impaired outflow), and disorders of optic nerve and visual pathways. Based on the preceding content and considering the actual clinical morbidity rate, our discussion will primarily focus on four key aspects.

### Disorders of choroid and retina

Our study identified a significant causal relationship between T2DM and disorders of choroid and retina, which is consistent with prior research findings. Previous studies have shown a risk of DR ranging from 50% to 60% in patients diagnosed with T2DM (22). The underlying mechanism of T2DM causing DR may be related to oxidative stress, resulting from the formation of advanced glycation end products (AGEs). Prolonged hyperglycemia can result in the non-enzymatic glycosylation of macromolecules, such as proteins and lipids, leading to a continuous increase in the levels of AGEs (23). Previous studies have demonstrated an association between AGEs and the prevalence of DR across all stages (24). The interaction between AGEs and their receptors on the cell surface activates nicotinamide adenine dinucleotide phosphate-oxidase, promoting the generation of intracellular reactive oxygen species (ROS) (25). In turn, the enhanced ROS levels contribute to the formation of AGEs, thereby exacerbating the damage caused by AGEs (26). Oxidative stress plays a pivotal role in the pathogenesis of diabetic retinopathy. The excessive accumulation of ROS can cause damage to the retinal endovascular and surrounding tissues, leading to the development of diabetic retinopathy (27).

### Disorders of optic nerve and visual pathways

The present study uncovered a potential positive causal relationship between T2DM and disorders of optic nerve and visual pathways. This finding is consistent with prior research. A cohort study found optic neuritis intensifies with the duration of diabetes and increasing glucose levels (28). This finding aligns with our research. The pathogenesis of optic nerve and visual pathway diseases in patients with T2DM may be attributed to hyperglycemia-induced oxidative stress. DM is characterized as an inflammatory disease that impacts the optic nerve through the release of inflammatory mediators resulting from oxidative stress (29). Moreover, previous research has demonstrated that elevated blood glucose levels induce the activation of Toll-like receptor 2 (TLR-2) and TLR-4 via reactive ROS, resulting in dysregulated microglial activation (30).

### Glaucoma

Our study identified a potential positive causal relationship between T2DM and glaucoma. However, a MR study conducted on an East Asian population found no statistically significant association between genetically predicted type 2 diabetes and POAG risk (31). This discrepancy may be attributed to ethnic differences. Nonetheless, a cohort study carried out in South Korea reported a significant association between the incidence of T2DM and open-angle glaucoma (32). Moreover, another cohort study conducted in Korea observed that T2DM was associated with a higher risk of glaucoma incidence (33). Furthermore, another MR study focusing on glaucoma revealed an independent and causal association between T2DM and glaucoma risk (34), which aligns with our own findings. Hyperglycemia may contribute to the increased accumulation of extracellular matrix (ECM) substances, which could be an underlying mechanism of glaucoma in individuals with diabetes. The abnormal accumulation of ECM substances is a key factor contributing to elevated intraocular pressure (35). The ECM primarily consists of fibronectin and glycosaminoglycans. Studies have demonstrated that elevated glucose levels can induce the synthesis of fibronectin in trabecular meshwork cells, which plays a crucial role in regulating aqueous outflow and intraocular pressure (36). Additionally, the stiffening of the cornea due to saccharification in individuals with diabetes also may compromise the flow of aqueous humor (37, 38).

### Senile cataracts

Our study has identified a significant causal relationship between T2DM and senile cataract, which is supported by previous research. A cohort study conducted in Sweden involving 35,369 women found that diabetic women had a 43% increased risk of cataract extraction (RR, 1.43; 95% CI, 1.10-1.86) (39). This finding is consistent with a meta-analysis of 20,837 subjects, which also confirmed T2DM as a risk factor for cataract (40). Additionally, Yuan et al. reported a positive correlation between genetic susceptibility to T2DM and senile cataract (41). Moreover, a previous MR study demonstrated that a higher genetic predisposition to T2DM was associated with an elevated risk of senile cataract (41). Kanishk et al. discovered that in diabetics, insufficient blood glucose control leads to increased aldose reductase (AR) levels and decreased glutathione (GSH) activity (42). AR is a critical initiator of sorbitol establishment in the lens, and abnormal sorbitol accumulation poses several risks to the lens, including lens opacity, interruption of the primary location for protein synthesis - the endoplasmic reticulum, ignition of apoptosis in lens epithelial cells, and oxidative stress damage to lens fibers (43–45). Additionally, GSH is one of the essential biochemical factors that maintain oxidative equilibrium in the lens; unusually low GSH levels can potentially affect lens transparency (46). Hence, the potential mechanism by which T2DM contributes to the development of senile cataracts may involve hyperglycemia-induced alterations in the activity of AR and GSH, thereby affecting lens metabolism.

### Strengths and limitations

A major strength of this study lies in its systematic analysis using MR to examine the causal relationships between T2DM and multiple ocular-related diseases. Furthermore, our findings were reinforced through rigorous sensitivity analyses, affirming the reliability and stability of our causal conclusions. Finally, by incorporating genetic variations, we minimized confounding interference, thereby upholding the validity of our study.

The MR method itself has inherent limitations and potential problems, such as weak instruments and pleiotropy. Despite our efforts to address these issues through various methods, there are still unavoidable challenges. Firstly, we need to consider the problem of population stratification, which refers to differences in disease incidence (or trait distribution) and allele frequencies among populations. Secondly, there is a possibility of bias due to sample overlap. Although we selected two distinct samples, it is important to note that the data from these samples may not be entirely independent, as both samples consist of European populations. Lastly, while we did not find evidence of horizontal pleiotropy, it is important to acknowledge the presence of residual bias since the precise function of most SNPs remains unknown. Lastly, since our study focused on the European population, the generalizability of our findings to other ethnic groups may be limited.

## Conclusion

In summary, the MR analysis results showed a significant positive causal relationship between T2DM and various ocular-related diseases, further demonstrating the adverse impact of diabetes on ocular health. As a corollary, addressing the necessity of maintaining adequate glycemic control becomes critical in protecting the ocular health of diabetics.

## Data availability statement

The original contributions presented in the study are included in the article/**Supplementary Material**. Further inquiries can be directed to the corresponding authors.

## Author contributions

RC: Writing - original draft. SX: Writing - original draft. YD: Writing - original draft. LL: Writing - original draft. CH: Writing - original draft. MB: Writing - review & editing. SL: Writing - review & editing. QW: Writing - review & editing.
