# Supplementary material for: Dissecting causal associations of type 2 diabetes with 111 types of ocular conditions: a Mendelian randomization study
Source: Front Endocrinol (Lausanne). 2023 Nov 22;14:1307468. doi: 10.3389/fendo.2023.1307468 (PMC10703475; doi:10.3389/fendo.2023.1307468)
Supplement: Supplementary file 1 [file DataSheet_1.docx]

**Supplementary Table 1**. Information of GWAS summary datasets used in MR analyses

| Traits | Data source | Phenotype ID | Sample size | Number of case | Number of control |
| --- | --- | --- | --- | --- | --- |
| Type 2 diabetes | Anubha Mahajan et al.（2018） | NA | 898130 | 74124 | 824006 |
| Acute and subacute iridocyclitis | IEU | finn-b-H7_IRIDOACUTE | 212413 | 3126 | 209287 |
| Acute inflammation of orbit | IEU | finn-b-H7_ORBITINFLAMACU | 203325 | 94 | 203231 |
| Age-related macular degeneration (whether dry or wet) | IEU | finn-b-H7_AMD | 209122 | 3763 | 205359 |
| Allergic conjunctivitis | IEU | finn-b-H7_ALLERGICCONJUNCTIVITIS | 218792 | 9833 | 208959 |
| Amblyopia ex anopsia | IEU | finn-b-H7_AMBLYANOPSIA | 211390 | 524 | 210866 |
| Anisometropia and aniseikonia | IEU | finn-b-H7_ANISOMETROPIA | 211293 | 362 | 210931 |
| Anomalies of pupillary function | IEU | finn-b-H7_ANOMAPUPFUNC | 217153 | 234 | 216919 |
| Aphakia | IEU | finn-b-H7_APHAKIA | 189829 | 225 | 189604 |
| Astigmatism | IEU | finn-b-H7_ASTIGMATISM | 211588 | 657 | 210931 |
| Atopic conjunctivitis | IEU | finn-b-H7_CONJUNCTIVITISATOPIC | 204099 | 582 | 203517 |
| Background retinopathy and retinal vascular changes | IEU | finn-b-H7_BCKRNDRETINOPAT | 203307 | 289 | 203018 |
| Blepharochalasis | IEU | finn-b-H7_BLEPHAROCHALASIS | 207366 | 4135 | 203231 |
| Blindness, monocular | IEU | finn-b-H7_BLINDMONOCULAR | 210959 | 93 | 210866 |
| Bullous keratopathy | IEU | finn-b-H7_BULLKERATOPATHY | 209387 | 100 | 209287 |
| Central retinal artery occlusion | IEU | finn-b-H7_CENTRRETARTOCC | 203269 | 251 | 203018 |
| Chalazion | IEU | finn-b-H7_CHALAZION | 205008 | 1777 | 203231 |
| Chorioretinal inflammation | IEU | finn-b-H7_CHORIORETINFLAM | 203402 | 384 | 203018 |
| Chronic conjunctivitis | IEU | finn-b-H7_CONJUNCTIVITISCHRON | 203924 | 407 | 203517 |
| Chronic iridocyclitis | IEU | finn-b-H7_IRIDOCHRONIC | 209982 | 695 | 209287 |
| Conjunctival degenerations and deposits | IEU | finn-b-H7_CONJUNCTDEGDEPOT | 203853 | 336 | 203517 |
| conjunctival haemorrhage | IEU | finn-b-H7_CONJUHAEMOR | 204359 | 842 | 203517 |
| Conjunctivitis | IEU | finn-b-H7_CONJUNCTIVITIS | 217172 | 13655 | 203517 |
| Conjunctivitis (acute, non atopic) | IEU | finn-b-H7_CONJUNCTIVITISACUNONATOPIC | 207709 | 4192 | 203517 |
| Convergence insufficiency and excess | IEU | finn-b-H7_CONVERGINSUFF | 211113 | 182 | 210931 |
| Convergent concomitant strabismus | IEU | finn-b-H7_CONVERSTRAB | 211898 | 967 | 210931 |
| Corneal degeneration | IEU | finn-b-H7_CORNEALDYSTROPHY | 209411 | 124 | 209287 |
| Corneal scars and opacities | IEU | finn-b-H7_CORNEALSCAR | 209551 | 264 | 209287 |
| Corneal ulcer | IEU | finn-b-H7_CORNULCER | 211887 | 2600 | 209287 |
| Crystalline deposits in vitreous body | IEU | finn-b-H7_VITRCRYSTAL | 211801 | 81 | 211720 |
| Dacryoadenitis | IEU | finn-b-H7_DACRYOADENITIS | 203333 | 102 | 203231 |
| Degenerated conditions of globe | IEU | finn-b-H7_GLOBEDEGENERATED | 211802 | 82 | 211720 |
| Degeneration of macula and posterior pole | IEU | finn-b-H7_MACULADEGEN | 209526 | 6508 | 203018 |
| Diplopia | IEU | finn-b-H7_DIPLOPIA | 212633 | 1767 | 210866 |
| Diseases of the eye and adnexa | IEU | finn-b-H7_EYE | 218792 | 75836 | 142956 |
| Dislocation of lens | IEU | finn-b-H7_LENSDISLOCATIO | 189792 | 188 | 189604 |
| Disorders of choroid and retina | IEU | finn-b-H7_CHOROIDRETINA | 218792 | 15774 | 203018 |
| Disorders of conjunctiva | IEU | finn-b-H7_CONJUNCTIVA | 218792 | 15275 | 203517 |
| Disorders of eyelid, lacrimal system and orbit | IEU | finn-b-H7_LIDLACRIMALORBIT | 218792 | 15561 | 203231 |
| Disorders of globe | IEU | finn-b-H7_GLOBE | 212048 | 328 | 211720 |
| Disorders of lacrimal system | IEU | finn-b-H7_LACRIMALSYSTEM | 209408 | 6177 | 203231 |
| Disorders of lens | IEU | finn-b-H7_LENS | 218792 | 29188 | 189604 |
| Disorders of ocular muscles, binocular movement, accommodation and refraction | IEU | finn-b-H7_OCUMUSCLE | 218792 | 7861 | 210931 |
| Disorders of optic nerve and visual pathways | IEU | finn-b-H7_OPTNERVE | 218792 | 1301 | 217491 |
| Disorders of orbit | IEU | finn-b-H7_ORBIT | 203855 | 624 | 203231 |
| Disorders of refraction and accommodation | IEU | finn-b-H7_REFRAACCOMMODIS | 214384 | 3453 | 210931 |
| Disorders of sclera, cornea, iris and ciliary body | IEU | finn-b-H7_SCLERACORNEA | 218792 | 9505 | 209287 |
| Disorders of vitreous body | IEU | finn-b-H7_VITRBODYGLOBE | 218502 | 6782 | 211720 |
| Disorders of vitreous body and globe | IEU | finn-b-H7_VITREOUS | 218792 | 7072 | 211720 |
| Divergent concomitant strabismus | IEU | finn-b-H7_DIVERGSTRAB | 212279 | 1348 | 210931 |
| Ectropion of eyelid | IEU | finn-b-H7_ECTROPION | 203704 | 473 | 203231 |
| Entropion and trichiasis of eyelid | IEU | finn-b-H7_ENTROPION | 204247 | 1016 | 203231 |
| Epiphora | IEU | finn-b-H7_EPIPHORA | 203509 | 278 | 203231 |
| Episcleritis | IEU | finn-b-H7_EPISCLERITIS | 209947 | 660 | 209287 |
| Exophthalmic conditions | IEU | finn-b-H7_EXOPTHALMUS | 203382 | 151 | 203231 |
| Fourth [trochlear] nerve palsy | IEU | finn-b-H7_PARASTRA4 | 211204 | 273 | 210931 |
| Glaucoma | IEU | finn-b-H7_GLAUCOMA | 218792 | 8591 | 210201 |
| Glaucoma secondary to eye inflammation | IEU | finn-b-H7_GLAUCSECINFLAM | 210297 | 96 | 210201 |
| Glaucoma secondary to eye trauma | IEU | finn-b-H7_GLAUCSECTRAUMA | 210296 | 95 | 210201 |
| Glaucoma suspect | IEU | finn-b-H7_GLAUCSUSP | 214967 | 4766 | 210201 |
| Glaucoma, exfoliation | IEU | finn-b-H7_GLAUCOMA_XFG | 211716 | 1515 | 210201 |
| Hereditary corneal dystrophies | IEU | finn-b-H7_KERATOCONUS | 209623 | 336 | 209287 |
| Hereditary retinal dystrophy | IEU | finn-b-H7_HEREDRETINADYST | 203221 | 203 | 203018 |
| Herpesviral keratitis and keratoconjunctivitis | IEU | finn-b-H7_HERPESKERATITIS | 209860 | 573 | 209287 |
| Heterophoria | IEU | finn-b-H7_HETEROPHORIA | 211992 | 1061 | 210931 |
| Hypermetropia | IEU | finn-b-H7_HYPERMETRO | 211717 | 786 | 210931 |
| Inflammation of lacrimal passages (chronic) | IEU | finn-b-H7_LACRIMINFLAMCHRON | 203470 | 239 | 203231 |
| Intermittent heterotropia | IEU | finn-b-H7_INTERHETEROTRO | 211754 | 823 | 210931 |
| Iridocyclitis | IEU | finn-b-H7_IRIDOCYCLITIS | 212909 | 3622 | 209287 |
| Keratitis | IEU | finn-b-H7_KERATITIS | 214848 | 5561 | 209287 |
| Keratoconus | IEU | finn-b-H7_CORNEALDEFORM | 209598 | 311 | 209287 |
| Lagophthalmos | IEU | finn-b-H7_LAGOPHTALMOS | 203381 | 150 | 203231 |
| Moderate visual impairment, binocular | IEU | finn-b-H7_MODVISIMPBINOC | 211281 | 415 | 210866 |
| Myopia | IEU | finn-b-H7_MYOPIA | 212571 | 1640 | 210931 |
| Normotensive glaucoma | IEU | finn-b-H7_GLAUCOMA_NTG | 211093 | 892 | 210201 |
| Ocular pain | IEU | finn-b-H7_OCUPAIN | 217812 | 893 | 216919 |
| Optic atrophy | IEU | finn-b-H7_OPTATROPHY | 217702 | 211 | 217491 |
| Optic neuritis | IEU | finn-b-H7_OPTNEURITIS | 218073 | 582 | 217491 |
| Paralytic strabismus | IEU | finn-b-H7_PARASTRAB | 211849 | 918 | 210931 |
| Peripheral retinal degeneration | IEU | finn-b-H7_PERIPHRETINADEGEN | 203428 | 410 | 203018 |
| Presbyopia | IEU | finn-b-H7_PRESBYOPIA | 211433 | 502 | 210931 |
| Primary angle-closure glaucoma | IEU | finn-b-H7_GLAUCCLOSEPRIM | 210789 | 588 | 210201 |
| Primary open-angle glaucoma | IEU | finn-b-H7_GLAUCPRIMOPEN | 214634 | 4433 | 210201 |
| Primary open-angle glaucoma, strict | IEU | finn-b-H7_GLAUCOMA_POAG | 213613 | 3412 | 210201 |
| Pterygium | IEU | finn-b-H7_PTERYGIUM | 203880 | 363 | 203517 |
| Ptosis of eyelid | IEU | finn-b-H7_PTOSIS | 204580 | 1349 | 203231 |
| Retinal breaks without detachment | IEU | finn-b-H7_RETINALBREAK | 205143 | 2125 | 203018 |
| Retinal detachment with retinal break | IEU | finn-b-H7_RETINALDETACHBREAK | 204953 | 1935 | 203018 |
| Retinal detachments and breaks | IEU | finn-b-H7_RETINALDETACH | 208584 | 5566 | 203018 |
| Retinal haemorrhage | IEU | finn-b-H7_RETINAHAEMORR | 203277 | 259 | 203018 |
| Retinal vascular disorders | IEU | finn-b-H7_RETINAVASC | 204660 | 1642 | 203018 |
| Retinal vascular occlusion | IEU | finn-b-H7_RETIVASCOCCLUSION | 204613 | 1595 | 203018 |
| Retinoschisis and retinal cysts | IEU | finn-b-H7_RETINOCHISISCYST | 203300 | 282 | 203018 |
| Scleritis | IEU | finn-b-H7_SCLERITIS | 209408 | 121 | 209287 |
| Scleritis and episcleritis | IEU | finn-b-H7_SCLERITISEPISCLERITIS | 210040 | 753 | 209287 |
| Senile cataract | IEU | finn-b-H7_CATARACTSENILE | 216362 | 26758 | 189604 |
| Separation of retinal layers (serosa) | IEU | finn-b-H7_RETINASEPAR | 203540 | 522 | 203018 |
| Severe visual impairment, monocular | IEU | finn-b-H7_SEVVISIMPMONOC | 210946 | 80 | 210866 |
| Sixth [abducent] nerve palsy | IEU | finn-b-H7_PARASTRA6 | 211319 | 388 | 210931 |
| Stenosis and insufficiency of lacrimal passages | IEU | finn-b-H7_STENOLACRI | 204549 | 1318 | 203231 |
| Strabismus | IEU | finn-b-H7_STRABISMUS | 218792 | 4620 | 214172 |
| Subjective visual disturbances | IEU | finn-b-H7_VISUDISTURBSUB | 214080 | 3214 | 210866 |
| Third [oculomotor] nerve palsy | IEU | finn-b-H7_PARASTRA3 | 211147 | 216 | 210931 |
| Traction detachment of retina | IEU | finn-b-H7_RETINALDETACHTRACTION | 203128 | 110 | 203018 |
| Use of antiglaucoma preparations and miotics | IEU | finn-b-H7_GLAUCOMAMEDICATION | 211428 | 1227 | 210201 |
| Vertical strabismus | IEU | finn-b-H7_VERTISTRAB | 211353 | 422 | 210931 |
| Visual disturbances | IEU | finn-b-H7_VISUDISTURB | 218067 | 7201 | 210866 |
| Visual disturbances and blindness | IEU | finn-b-H7_VISUALDISTBLIND | 218792 | 7926 | 210866 |
| Visual field defects | IEU | finn-b-H7_VISFIELDDEF | 212044 | 1178 | 210866 |
| Visual impairment including blindness (binocular or monocular) | IEU | finn-b-H7_BLINDANDVISIMPAIRMENT | 211769 | 903 | 210866 |
| Vitreous haemorrhage | IEU | finn-b-H7_VITRHAEMORR | 213085 | 1365 | 211720 |
| Xanthelasma of eyelid | IEU | finn-b-H7_XANTHELASMA | 203342 | 111 | 203231 |
| Abbreviation: GWAS: genome-wide association study; MR: Mendelian randomization; NA: Not Applicable. | | | | | |

**Supplementary Table 2**. The range of F-statistics of IVs

|  | F-Statistic |
| --- | --- |
| Maximal | 1393.78 |
| Average | 75.82 |
| Median | 47.27 |
| Minimal | 29.47 |

**Supplementary Table 3**. MR analysis results

| Outcome | Method | Number of SNPs | OR | 95%CI | *P* |
| --- | --- | --- | --- | --- | --- |
| Diseases of the eye and adnexa | IVW | 184 | 1.05 | 1.03~1.08 | 3.80E-05 |
| Diseases of the eye and adnexa | MR Egger | 184 | 1.05 | 1.00~1.11 | 6.12E-02 |
| Diseases of the eye and adnexa | WM | 184 | 1.04 | 1.01~1.07 | 4.38E-03 |
| Disorders of choroid and retina | IVW | 184 | 1.13 | 1.06~1.19 | 3.34E-05 |
| Disorders of choroid and retina | MR Egger | 184 | 1.16 | 1.03~1.31 | 1.72E-02 |
| Disorders of choroid and retina | WM | 184 | 1.11 | 1.05~1.17 | 8.26E-05 |
| Senile cataract | IVW | 184 | 1.07 | 1.03~1.11 | 7.77E-04 |
| Senile cataract | MR Egger | 184 | 1.05 | 0.97~1.14 | 2.28E-01 |
| Senile cataract | WM | 184 | 1.07 | 1.01~1.13 | 1.98E-02 |
| Abbreviation: MR: Mendelian randomization; OR: odds ratio; CI: confidence interval; SNP: single nucleotide polymorphism; IVW: inverse-variance weighted; WM: weighted median. | | | | | |

**Supplementary Table 4.** Heterogeneity test results

| Outcome | Q | Q_df | Q_*P* |
| --- | --- | --- | --- |
| Diseases of the eye and adnexa | 316.65 | 183 | 3.23E-09 |
| Disorders of choroid and retina | 571.15 | 183 | 1.71E-41 |
| Senile cataract | 292.68 | 183 | 4.64E-07 |

**Supplementary Table** **5**. The pleiotropy analyses

| Outcome | Egger intercept | *SE* | *P* |
| --- | --- | --- | --- |
| Diseases of the eye and adnexa | -2.24E-05 | 0.002 | 9.90E-01 |
| Disorders of choroid and retina | -2.40E-03 | 0.004 | 5.63E-01 |
| Senile cataract | 1.00E-03 | 0.003 | 7.21E-01 |
| Abbreviation: SE: standard error. | | | |

**Supplementary Table 6.** MR-PRESSO analyses

| Outcome | MR Analysis | Causal Estimate | SD | *P*-value |
| --- | --- | --- | --- | --- |
| Diseases of the eye and adnexa | Raw | 0.05 | 0.01 | 5.75E-05 |
|  | Outlier-corrected | 0.04 | 0.01 | 1.02E-04 |
| Disorders of choroid and retina | Raw | 0.12 | 0.03 | 5.11E-05 |
|  | Outlier-corrected | 0.09 | 0.02 | 2.17E-06 |
| Senile cataract | Raw | 0.06 | 0.02 | 9.46E-04 |
|  | Outlier-corrected | 0.06 | 0.02 | 8.27E-04 |
| Abbreviation: MR: Mendelian randomization; SD: standard deviation. | | | | |


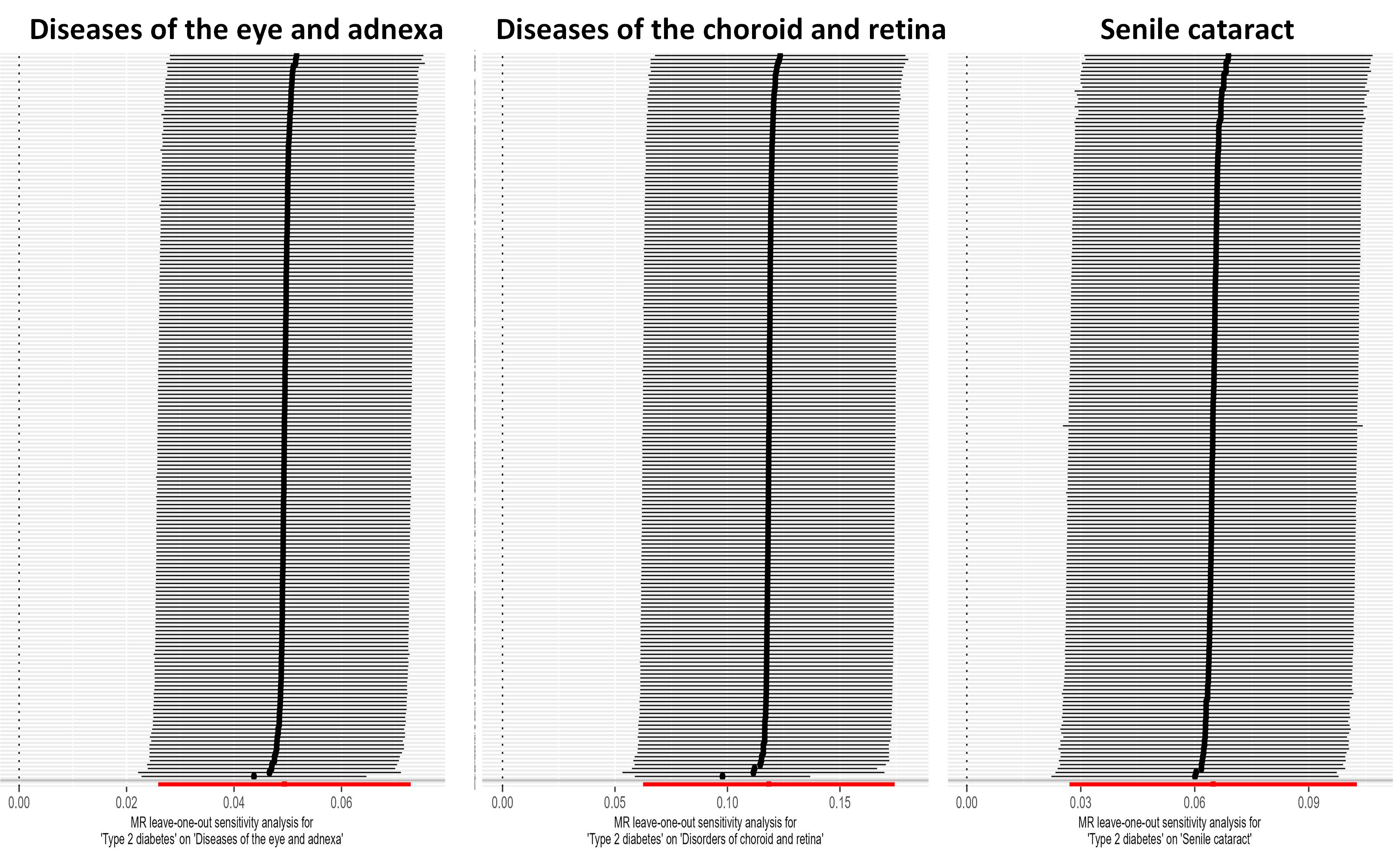


**Supplementary Figure 1**. Leave-one-out sensitivity analysis examining the causal estimates of type 2 diabetes mellitus on ocular-related diseases by the IVW method after exclude a specific SNP from the analysis. The red line represents the IVW estimate of all SNPs on outcome. MR: Mendelian randomization; SNP: single nucleotide polymorphism; IVW: inverse-variance weighted.
